# Prevalence of chronic non-specific low back pain among caregivers of stroke survivors in Kano, Nigeria and factors associated with it: A cross-sectional study

**DOI:** 10.3389/fneur.2022.900308

**Published:** 2022-10-05

**Authors:** Auwal Abdullahi, Kamilu Aliyu, Auwal Bello Hassan, Ganiyu Oluwaleke Sokunbi, Bashir Bello, Wim Saeys, Steven Truijen

**Affiliations:** ^1^Department of Physiotherapy, Bayero University Kano, Kano, Nigeria; ^2^Department of Medical Rehabilitation (Physiotherapy), College of Medical Sciences, University of Maiduguri, Maiduguri, Nigeria; ^3^Department of Rehabilitation Sciences and Physiotherapy, University of Antwerp, Antwerp, Belgium

**Keywords:** stroke, low back pain, disability, caregivers, quality of life, activities of daily living, Nigeria, cross-sectional study

## Abstract

**Purpose:**

Low back pain (LBP) may have a specific or non-specific cause such as abnormal posture or repetitive tasks. For instance, lifting and transferring patients during caregiving for stroke survivors may predispose the caregivers to LBP.

**Objectives:**

The aim of this study is to determine the prevalence of chronic non-specific LBP and factors associated with it in caregivers of stroke survivors.

**Method:**

The research design used is cross-sectional study design. Participants of the study were caregivers of stroke survivors in Kano, Nigeria who were at least 18 years old. They were included if they had at least one-month experience with caregiving for at least 1 h per day. Presence of LBP and level of disability were assessed using participants' self-report and Rolland Morris Low Back Pain Disability Questionnaire respectively. The data collected was analyzed using descriptive, Chi-square statistics and Binary Logistics Regression.

**Result:**

Three hundred caregivers with mean age, 33.24 ± 10.32 years in which 207 and 93 were males and females respectively, participated in the study. The results showed that, there was a high prevalence (64.7%) of LBP among the caregivers. The prevalence was significantly associated with gender (*p* < 0.001), age (*p* = 0.029), occupation (*p* < 0.001) and duration of caregiving (*p* < 0.001) of the study participants. In addition, the result of the regression model showed that, being a female (*p* = 0.001), a civil servant (*p* = 0.031), a trader (*p* = 0.013), and a complete caregiver (0.001); and caregiving for a duration of 5 h or more per day (*p* = 0.024) are significant predictors of having LBP. Similarly, level of disability due to the presence of LBP among the study participants was significantly associated with gender (*p* < 0.001), occupation (*p* < 0.001), duration of caregiving (*p* = 0.025), and the nature of the caregiving (*p* < 0.001).

**Conclusion:**

Informal caregiving for stroke survivors may result in developing chronic non-specific LBP, especially among females, Civil servants, traders, complete caregivers and those with long duration of caregiving. This can add an additional burden on the family in terms of cost of care, result in reduced quality of caregiving and cause psychological stress. Thus, it is important the health of the caregivers of stroke survivors is considered during stroke rehabilitation.

## Introduction

Low back pain (LBP) is an important public health concern. It is a pain experienced at the lower part of the vertebral spine known as the lumbar spine (1, 2); and it is one of the leading causes of a patient visit to emergency department (3). It may have or may not have a specific cause, and thus it can be classified as specific or non-specific type of LBP (4). The specific type of LBP is the one that is attributable to a specific pathology such as radiculopathy, disc herniation, spondylolisthesis, lumbar spine stenosis, osteoporosis and scoiliosis (5). The non-specific type of LBP is the one that is not attributable to any specific pathology (6).

Prevalence of occupation-based LBP among those who are aged 19 to 64 years in Kano, Nigeria was estimated to be between 32.5 and 73.5% (7, 8). Additionally, it is associated with a working posture. The pain may cause considerable burden on individuals, families, and communities (9). Other factors that are known to be associated with LBP include sex, age, marital status, attitude about a healthy life style, physical activity involving work and tobacco smoking (1). The work-related factors that are highly associated with LBP include those involving heavy weight lifting, and those that can cause abnormal posture in sitting, standing, and walking (7, 10).

Caregiving is the act of providing physical and emotional support for an ill or disabled individual which could be a child, spouse, friend or any member of the family or the healthcare providers such as the nurses and the physiotherapists. It is divided into formal and informal caregiving. Formal caregiving is provided by the healthcare providers such as the nurses, the physiotherapists and the physicians (11); whereas, informal caregiving is provided by people who are unpaid for it, such as the members of the patient's family, friends and neighbors (12). However, in low and middle income countries, access to formal care is limited, largely due to factors such as low capacity of the health facilities, limited number of the facilities, distance from homes to the centers and cost of care (13).

Following stroke, the informal caregivers engage in helping the patients with both basic and instrumental activities of daily living (ADL) such as physical movement, personal hygiene, facilitating transfer, managing finances, provision of healthy nutrition, and facilitation of any needed healthcare and prevention of other complications (14). In the process of helping patients with performance of functional activities such as lifting and transfer, caregivers may be at the risk of developing musculoskeletal symptoms such as LBP due to repeated physical task or poor ergonomics (15). Presence of LBP among caregivers can affect the quality of the caregiving, and stroke survivours whose caregivers had LBP showed a significantly lower recovery in ability to carrying out activities of daily living (16).

Thus far, there seems to be only two studies that specifically looked at the prevalence of LBP in informal caregivers of stroke survivours (16, 17). The first study was carried out in Bangladesh, where one of the risk factors of LBP could be smoking habit and anthropometric characteristics such as height (18, 19). Smoking is associated with the risk of having LBP (1, 20). Similarly, height is one of the variables used to estimate body mass index (BMI), and increasing BMI is a risk factor for LBP (21). However, in Kano, Nigeria, smoking and anthropometric characteristics of the people may not be similar to those in Bangladesh. In addition, in the second study, only 64 participants were included in the study (16), which a sample too small to provide a reliable information.

Consequently, it is important to further evaluate the prevalence of LBP among caregivers of patients with stroke in this population. This is important as there is currently increase in the prevalence of stroke in developing countries such as Nigeria (22), where informal caregivers play significant roles in managing the patients (16). In addition to the above, LBP causes significant economic burden especially as related to the cost of physiotherapy and other medical care (9, 23). Therefore, the aim of this study is to determine the prevalence of LBP among caregivers of patients with stroke in Kano, Nigeria and the factors associated with it. We hypothesized that LBP would be highly prevalent among the targeted population and it will be significantly associated with the characteristics of the participants, as well as complete and longer duration of caregiving.

## Method

This study was a cross-sectional study. The participants were 300 caregivers of stroke survivors attending Physiotherapy departments at Murtala Muhammed Specialists Hospital, Muhammad Abdullahi Wase Specialist Hospital, and Aminu Kano Teaching Hospital, in Kano, Nigeria. Overall, participants were included in the study if they provided care for at least one-month duration to a stroke survivor who, at least, needed help with activities of daily living and without any history of LBP prior to the start of the caregiving or back related musculoskeletal surgical procedure. The participants were recruited using convenience sampling technique. Ethical approval was obtained from the Aminu Kano Teaching Hospital (AKTH) Health Research Ethics committee, and the Research Ethics committee of Kano State Hospital Services Management Board. Participants' informed consent was also obtained.

### Sample size estimation

The estimated sample size for the study was 316 caregivers of stroke survivors. The sample size was calculated using the formula, $n=\frac{Z^{2}p\left(1-p\right)}{d^{2}}$ according to Daniel (16). Where z = level of confidence = 1.96 at 95% CI, *p* = expected prevalence = 71% [taken from a study by Salma (17)] and d = precision (Corresponding to effect size) = 5%.

### Outcome measurement

The study data was collected using a proforma and Rolland Morris low back pain disability questionnaire (RMDQ) administered by trained research assistants. The proforma contained socio-demographic information such as gender, age, education level, occupation, height, weight, BMI of the caregiver, duration of daily caregiving in hours and the nature of caregiving tasks. The Rolland Morris low back pain disability questionnaire (RMDQ) was used to assess the level of disability resulting from the LBP. The questionnaire has good content and construct validity in assessing LBP (24), concurrent validity with Oswestry disability index questionnaire (25), and test-retest reliability (26). The questionnaire has a total score of 24 which can be categorized as 0 = no disability, 1–8 = mild disability, 9–16 = moderate disability and 17–24 = severe disability.

For the presence of LBP, participants' self-report of whether they had any discomfort in their lower back (such as numbness, ache, and pain) at any time during the last 12 months was recorded. In addition, RMDQ was used to assess disability where a score of 0 is deemed absence of disability or low back pain. A score of 1–24 is considered presence of low back pain since back pain is associated with disability. Thus, if a participant answered 'yes' to the self-report, they were then asked further about the disability related to the LBP using RMDQ. The study data was collected by blinded (from the aim of the study) trained research assistants between 1st June, 2018 and 28^th^ August, 2018.

### Data analysis

Descriptive statistics was used to summarize the characteristics of the study participants and to estimate the prevalence of LBP in the caregivers using percentage. Additionally, Chi-square was used to determine the association between prevalence of LBP and characteristics of the participants. Furthermore, binary logistic regression was used to determine the likelihood of the characteristics of the participants to predict presence of low back pain. All analyses were performed at 0.05 level of significance using statistical packages for social sciences (SPSS) version 20.

## Results

### Demographic characteristics of the study participants

**Figure 1:**
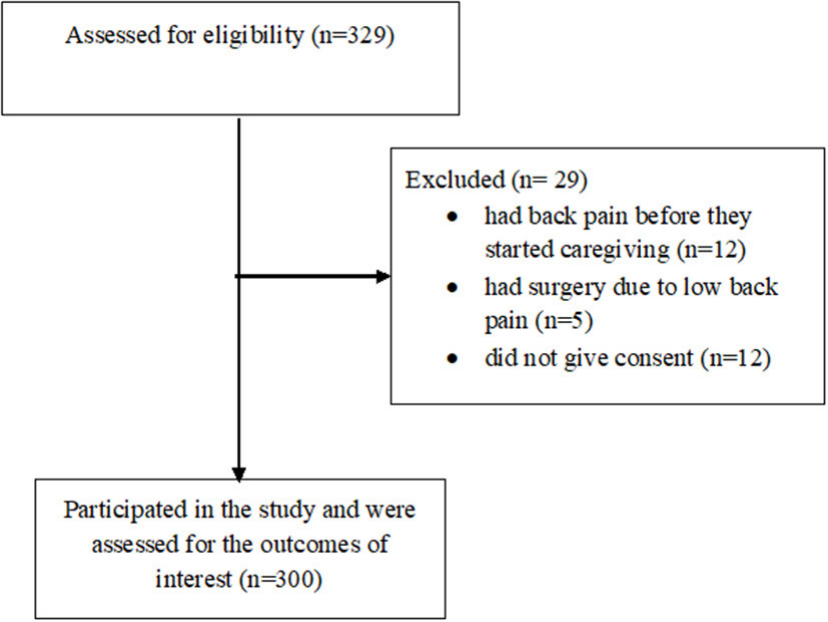
The study flowchart.

**Figure 2:**
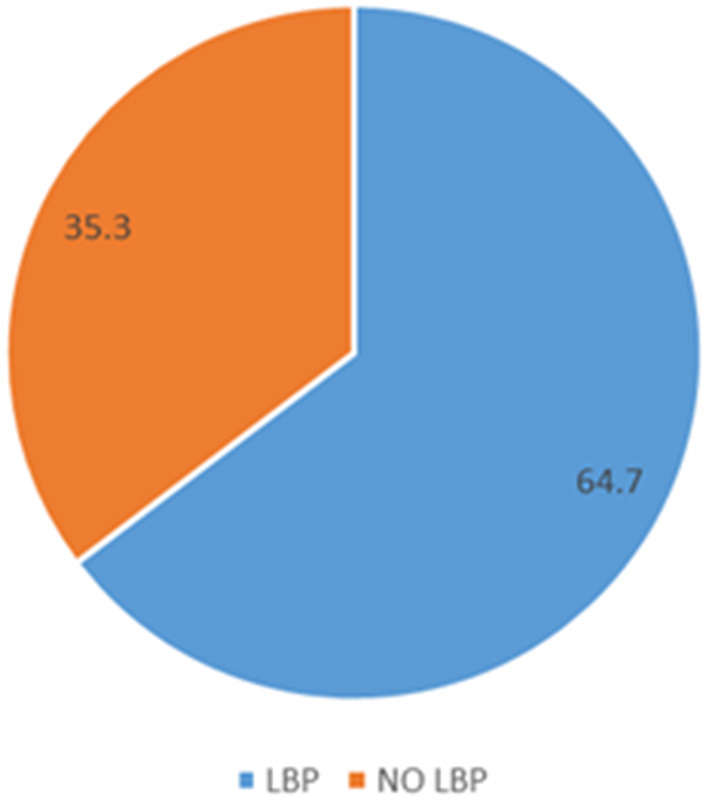
Pie chart representing the prevalence.

Initially, 329 participants were recruited. However, 12 had low back pain before they started caregiving, 5 had surgery due to low back pain and 12 others did not give consent citing that they are staff of the hospital (see Figure 1 for the study flowchart). They were thus excluded from the study. Consequently, 300 caregivers of stroke survivors participated in the study with age range between 19 and 60 years (mean age, 33.24 ± 10.32 years). Out of this number, majority, 207 (69.0%), 132 (44.0%), 171 (56.8%) were men, traders and married respectively. Details of the demographic characteristics of the study participants are presented in Table 1. The prevalence of LBP among caregivers of the stroke survivors is 64.7%. See Figure 2 for the pie chart representing the prevalence.

**Table 1:** Characteristics of the study participants.

**Variables**	***n***	**%**
Presence of LBP (Yes/No)	194/106	64.7/35.3
Gender (M/F)	207/93	69.0/31.0
Age (1/2/3/4)	130/103/45/22	43.0/34.3/15.0/7.3
Marital Status (M/S)	171/129	56.8/42.9
Educational Status (N/P/S/T)	88/24/112/75	29.2/8.0/37.2/25.0
Occupation (C/T/S/L/H/F)	42/132/34/33/52/7	14.0/44.0/11.3/11.0/17.3/2.3
Duration of caregiving (1/2/3/4)	233/11/6/28	77.7/11.0/2.0/9.3
Disability Category (Mild/Moderate/Severe)	81/8/211	27/2.7/70.3
Nature of caregiving (Complete /Partial)	225/75	75.0/25.0

LBP, Low back pain; M/F, Male/female; Age 1/2/3/4, 19–29/30–40/41–50/51–60; M/S, Married/Single; N/P/S/T, No formal/Primary/Secondary/Tertiary; C/T/S/L/H/F, Civil servant/Trader/Student/Laborer/Housewife/Farmer; Duration of caregiving (1/2/3/4), 1–5/6–10/11–15/>15 h.

### Association between presence of LBP and the characteristics of the caregivers of stroke survivors

For gender, there was a significant association between presence of LBP and gender (*p* < 0.001, phi = −0.304). The result indicates that, LBP is more prevalent in male (79.4%) than female (20.6%) caregivers. For age, there was no significant association between presence of LBP and age (*p* > 0.05, phi = −0.096). However, the result indicates that, LBP is more prevalent in the age category, 19–29 (43.3%) than 30–40 (32.0%), 41–50 (6.0%) and 51–60 (8.8%) years.

For marital status, there was no significant association between presence of LBP and marital status (*p* > 0.05, phi = −0.062). However, the result indicates that, LBP is more prevalent in the singles (59.4%) than married (40.7%). For educational status, there was no significant association between presence of LBP and educational status (*p* > 0.05, phi = −0.078). However, the result indicates that, LBP is more prevalent in those with secondary level education, 19–26 (37.6%) than those with no formal education (27.8%), tertiary education (25.8%) and primary education (7.7%). For occupation, there was a significant association between presence of LBP and occupation (*p* < 0.001, phi = −0.383). However, the result indicates that, LBP is more prevalent in traders (51.0%) than laborers (14.4%), students (12.4%), civil servants (10.8%), housewives (8.2%) and farmers (3.1%).

For body mass index (BMI), there was no significant association between presence of LBP and BMI (*p* > 0.05, phi = −0.121). However, the result indicates that, LBP is more prevalent in those with normal BMI (58.8%) than those who are overweight (18.6%), underweight (18.0%), and obese type 1 (3.1%) and obese type II (1.5%). For daily duration of caregiving, there was a significant association between presence of LBP and daily duration of caregiving (*p* < 0.05, phi = 0.171). However, the result indicates that, LBP is more prevalent in those with caregiving duration of 1–5 h (76.8%), than those with >15 h (12.4%), 6–10 h (9.8%), and 11–15 h (1.0%). For nature of caregiving, there was significant association between presence of LBP and nature of caregiving (*p* < 0.001, phi = −0.217). However, the result indicates that, LBP is more prevalent in those with partial caregiving (82.0%) than those with complete caregiving (18.0%). Details of this result are presented in Table 2.

**Table 2:** Association between presence of LBP among caregivers of stroke survivors and demographic and clinical variables.

**Variables**	**X^2^**	***P***
Gender	26.31	<0.001
Age	2.78	0.409
Marital status	0.915	0.339
Educational status	1.848	0.764
Occupation	44.034	<0.001
BMI	4.421	0.352
Daily duration of caregiving	13.613	0.011
Nature of caregiving	13.149	0.001

BM, Body mass index; n, number of the study participant; d/f, degree of freedom; x^2^, Chi-square; *p*, level of significance.

### Association between level of disability and participants' characteristics

For level of disability, there was only significant association between level of disability and participants' characteristics such as gender (X^2^ = 24.805, *P* < 0.001), age (X^2^ = 14.02, *P* = 0.029), occupation (X^2 =^ 58.917, *P* = 0.001), and nature of caregiving (X^2^ = 12.619, *P* = 0.002). See Table 3 for the details of the result.

**Table 3:** Association between Level of disability and Participants' Characteristics (N300).

**Characteristics**	**Mild disability (%)**	**Moderate disability (%)**	**None (%)**	**X^2^**	***P***
**Gender**				24.805	<0.001*
Male	19.8	1.0	79.2		
Female	41.9	8.6	49.5		
**Age (years)**				14.02	0.029*
19–29	23.8	1.5	74.6		
30–40	35.0	2.9	62.1		
41–50	17.8	11.1	71.1		
51–60	22.7	0.0	77.4		
**Educational status**				8.108	0.230
No formal education	29.5	3.4	67.0		
Primary	8.0	8.0	84.0		
Secondary	26.8	3.6	69.6		
Tertiary	29.3	1.3	69.3		
**Occupation**				58.917	0.001*
Civil servant	38.1	7.1	54.8		
Trader	17.4	0.8	81.8		
Student	20.6	0.0	79.4		
Laborer	12.1	0.0	87.9		
Housewife	55.8	11.5	32.7		
Farmer	14.3	0.0	85.7		
**Daily Duration of caregiving**				26.627	<0.001*
1–2 h	25.0	1.0	61.3		
3–4 h	0.7	1.7	8.7		
5 h and above	1.3	0.0	0.3		
**Nature of caregiving**				12.619	0.002*
Partial caregiving	21.8	2.7	75.6		
Complete caregiving	41.3	5.3	53.3		
**BMI**				8.622	0.163
Underweight	4.0	0.3	13.3		
Normal weight	14.0	1.0	42.7		
Overweight	8.0	1.3	12.7		
Obese	1.0	0.0	1.7		

^*^Statistically significant.

### Predictors of likelihood of presence of LBP among caregivers of stroke survivours

The model contained 4 independent variables (gender, occupation, duration of caregiving, and nature of caregiving). The full model containing all predictors was statistically significant, x^2^ (10, *N* = 300) = 80.617, *p* < 0.001, indicating that the model was able to distinguish between participants who have LBP and those who do not have. The model as a whole explained between 23.66% (Cox and Snell R square) and 32.4% (Negelkerke R square) of the presence or absence of LBP, and did correctly classify 60.4% of cases.

In addition, the result showed that, only being a female (*p* = 0.001), a civil servant (*p* = 0.031), a trader (*p* = 0.013), and a complete caregiver (0.001); and caregiving for a duration of 5 hours or more per day (*p* = 0.024) significantly predicted presence low back pain. See Table 4 for the details results.

**Table 4:** Predictors of likelihood of presence of low back pain among caregivers of stroke survivours.

**Variable**	**B**	**S.E**	**Walid**	**df**	**Odd ratio**	**95% CI**		**p**
						**Lower limit**	**Upper limit**	
**Gender** (female)	−1.271	0.375	11.490	1	0.3	0.1	0.6	0.001*
**Occupation**								
Civil servant	0.875	0.405	4.661	1	2.4	1.1	5.3	0.031*
Trader	1.425	0.575	6.135	1	4.2	1.3	12	0.013*
Student	2.031	0.638	10.147	1	7.6	2.2	27	0.001*
Laborer	−0.269	0.531	0.257	1	0.8	0.3	2.2	0.612
Housewife	2.266	1.387	2.671	1	9.6	0.6	146	0.102
**Duration of caregiving**								
1 to 2 h	−0.105	0.444	0.056	1	0.9	0.4	2.1	0.813
3 to 4 h	0.072	1.039	0.005	1	1.1	0.1	8.2	0.945
5 h and above	1.478	0.655	5.085	1	4.4	1.2	16	0.024*
**Nature of caregiving** (complete)	−1.155	0.336	11.822	1	0.3	0.2	0.6	0.001

^*^Statistically significant.

## Discussion

The main aim of the study was to determine the prevalence of LBP among caregivers of stroke survivors and the factors associated with it. The result showed that, the prevalence was high. In addition, there was significant association between presence of LBP and gender, occupation, duration of caregiving and nature of caregiving. Similarly, there was significant association between level of disability and gender, age category, occupation, daily duration of caregiving and nature of caregiving. Although, the present study reported a relatively high prevalence of LBP, previous studies reported much higher prevalences of the condition, 71 and 82.8% respectively (16, 17). However, the study by Yalchikanya and colleagues was on inpatients stroke survivours (16). Inpatient stroke survivors are mostly within the early stage post stroke and as such they may need more assistance with ADL especially lifting and transferring. Thus, it is not surprising if the study reported a higher prevalence than the present study. Similarly, in the study by Salma (17), the setting was Bangladesh, where smoking habit was estimated to be about 26% (18); and smoking itself, is a risk factor for LBP (27).

Additionally, anthropometric characteristics such as height may predispose someone to develop LBP (20). Probably there is height difference between Bangladeshis and our population. Height is one of the variables used to estimate BMI, and increasing BMI is a risk factor for LBP (21). Therefore, these two aforementioned reasons could be responsible for higher prevalence of LBP among Bangladeshi caregivers than the caregivers in Kano. Moreover, there are other studies that investigated the prevalence of musculoskeletal injuries among caregivers of stroke survivors where LBP emerged top. For instance, Hayes et al. (28) conducted a study to determine the prevalence of physical injuries and their types among 275 caregivers of veterans with stroke. They found LBP to be the most prevalent, constituting 56% of the total injuries among the 66 injured caregivers. Similarly, another study conducted in Maiduguri city in Nigeria reported LBP to be the most prevalent with a prevalence of 72.2% (29). In agreement with the findings from the population of caregivers of stroke survivours, similar findings have been reported among caregivers of elderly (30–32).

Nevertheless, caregiving for stroke survivours may be influenced by the family setting and culture of the stroke survivors. For instance, in the presence study, there is a significant association between presence of LBP and gender with a higher prevalence in males than in females. This is unlike in a previous study where there was no significant association between presence of LBP and gender of the study participants (17). The difference between these two studies could be because of the sample sizes of the two studies and the ratio of females to males. This is because, in Bangaladesh, according to the authors females are given more responsibilities (17); thus, the likelihood of finding association between gender and presence of LBP is expected to be high. In contrast, in Kano, Nigeria, responsibility can be given to either gender depending on the need. Hence in the present study, the number of males was more than twice the number of females. Furthermore, the participants of this study were recruited from the relatives accompanying stroke survivors that attend physiotherapy departments on out-patient basis. Accompanying stroke survivours for physiotherapy in this community is usually done by males since it is considered to be physically demanding. Thus, the males are more likely to have LBP.

In addition, male gender preponderence in this study could be behind the non-significant association between presence of LBP and marital stutus of the caregivers. Previous studies reported spousal intimacy to be one of the predisposing factors in male caregivers, as it provides strong emotional motivation, making them work beyond their physical capacity and often overlook their back discomfort (27). Similarly, in Nigerian culture, caregiving activities are mostly carried out by informal family caregivers which include the spouses and children of the stroke survivors. As explained earlier, children of the survivors often accompany them to the clinic which increase their chances of being recruited into this study. Therefore, it is not surprising that, caregivers who are in the age categories, 19–29 and 30–40 years have highier prevalence of LBP, 43.3 and 32.0% respectively. This finding is supported by prevoius studies conducted in Nigeria in which the caregivers were predominantly the children of the survivors (33, 34). In contrast, in western contries, caregivers are mainly the spouses (28–36). Consequently, it is important to properly train caregivers of stroke survivours on the arts of caregiving in order to help safeguard their health and prevent LBP.

Another important finding of this study is that, there was significant association between presence of LBP and duration of caregiving. Suprisingly, the prevalence is higher (76.8%) during the shortest daily duration of caregiving (1–5 h). However, previous studies found no significant association between presence of of LBP and duration of caregiving (17, 28). In addition, perceived burden has been reported to have positive correlation with duration of caregiving among caregivers of stroke survivours (37). Consequently, since in the present study, most of the participants spend less hours for caregiving, it is possible that their LBP might be caused by other activities they engage in such as their occupation (38). Evidently from the present study, occupation was significantly associated with presence of LBP, albeit, with traders having the highest prevalence (51.0%) and farmers with the least (3.1%). Occupations with increase in physical demand, repetitive motion and assumption of a particular posture for a long duration, have higher risks for LBP (39). In particular, trading is among the leading predisposing factors for LBP as revealed in a previous study conducted in Enugu, Nigeria (40).

In contrast, a study in Ibadan, Nigeria reported a higher prevalence of LBP (55%) among farmers (41). This difference may be related to the nature of the caregiving. For instance, both Kano, where the present study was conducted and Enugu are among the major economic capitals of Nigeria with significant number of their populations being involved in various kinds of trading occupation. Traders are predisposed to repetitive trauma to their low back as a result of lifting of heavy objects and long daily duration of sitting posture. In addition, some of the caregiving activities such as lifting are also very demanding and as such in the absence of lack of proper knowledge about the techniques of handling patients, LBP could develop (42). Perhaps, this could be the reason for the significant association between nature of caregiving and presence of LBP in this study, although those providing partial caregiving have higher prevelence (82%) compared to the complete caregivers (18%). However, a study conducted in Thailand reported full time caregivers to have slightely higher odd ratio (1.67) of developing LBP compared to partial caregivers (1.36) (43). The difference between our finding and their own can be explained by the activities carried out by the caregivers during the caregiving since presence of LBP may be associated with the type of the caregiving activity.

Finally, concerning the level of disability due to LBP and its association with the participants' characteristics, our study revealed significant association between it and gender, age, occupation, duration of caregiving and the nature of caregiving. Although most of the participants had no disability, 55.8% of those who are housewives have mild disability. This could be attributed to the involvement of females in indoor activities of bending and extending the lower back such as during household chores (44). Therefore, the findings of this study highlight the need for more inclusive rehabilitation approach that will be patient-caregiver centered as opposed to the patient-centered approach only that is currently being practiced or advocated (45, 46). An example of such approach, called Supportive Educative Learning Programme for family caregivers (SELF), was developed and implemented in Thailand with great success (47). Thus, similar physiotherapist-led program should be developed in Nigeria to address caregivers' health needs.

One of the strength of this study is the cross-sectional nature of surveying 300 caregivers of patients with stroke. Large sample size is important in attributing the case to the population (48). However, a weakness of special note in the study is the reliance on participants' self-report of having back pain. Self-reports can be very subjective (49). In addition, we did not record whether the participants sought for any clinical attention and the time it took them to do so from the onset of the symptom. Consequently, we urge future studies to observe and follow up caregivers of stroke survivours from the beginning of the caregiving process using both objective and patients' reported outcomes for back pain.

## Conclusion

Chronic non-specific LBP is common among caregivers of patients with stroke especially among males, traders, complete caregivers and those with long duration of caregiving. Therefore, it is important for rehabilitation clinicians to educate caregivers on proper patient's lifting and transferring techniques in order to help prevent occurrence of LBP. This is because informal caregivers are important partners in the rehabilitation of patients with stroke, and their health is essential to effective provision of caregiving services.

## Data availability statement

The raw data supporting the conclusions of this article will be made available by the authors, without undue reservation.

## Ethics statement

The studies involving human participants were reviewed and approved by Aminu Kano Teaching Hospital (AKTH) Health Research Ethics Committee, and the Research Ethics Committee of Kano State Hospital Services Management Board. The patients/participants provided their written informed consent to participate in this study.

## Author contributions

AA and KA conceived the idea and analyzed the data. ABH and AA drafted the manuscript. All authors contributed to the study design, interpretation of the result, critical review of the manuscript, and approved the manuscript for publication.

## Conflict of interest

The authors declare that the research was conducted in the absence of any commercial or financial relationships that could be construed as a potential conflict of interest.

## Publisher's note

All claims expressed in this article are solely those of the authors and do not necessarily represent those of their affiliated organizations, or those of the publisher, the editors and the reviewers. Any product that may be evaluated in this article, or claim that may be made by its manufacturer, is not guaranteed or endorsed by the publisher.

## References

[1] Manniche C Lundberg E Christensen I Bentzen L Hesselsøe G. Intensive dynamic back exercises for chronic low back pain: a clinical trial. Pain. (1991) 47:53–61. 10.1016/0304-3959(91)90011-L 1837606

[2] Ralston SH Walker BR. Colledge NR. Davidson's Principle and Practice of Medicine (21^*st*^ ed). London: Elsevier Health Sciences (2010) p. 1180–90.

[3] Edwards J Hayden J Asbridge M Gregoire B Magee K. Prevalence of low back pain in emergency settings: a systematic review and meta-analysis. BMC Musculoskelet Disord. (2017) 18:143. 10.1186/s12891-017-1511-7 28376873 PMC5379602

[4] Suzuki H Kanchiku T Imajo Y Yoshida Y Nishida N. Diagnosis and characters of non-specific low back pain in Japan: The yamaguchi low back pain study. PLoS ONE. (2016) 11:e0160454. 10.1371/journal.pone.0160454 27548658 PMC4993356

[5] Balagué F Mannion AF Pellisé F Cedraschi C. Non-specific low back pain. Lancet. (2012) 379:482–91. 10.1016/S0140-6736(11)60610-7 21982256

[6] Koes BW Van Tulder MW. Clinical Review, Diagnosis and treatment of low back pain. BMJ. (2006) 332:1430. 10.1136/bmj.332.7555.1430 16777886 PMC1479671

[7] Ahmad RY Elmi OS Aliyu SU Jajere AM Digil AA. Prevalence and risk factors for low back pain among professional drivers in Kano, Nigeria. Arch Environ Occup Health. (2015) 70:251–5. 10.1080/19338244.2013.845139 24219691

[8] Bello B Bello Adebayo H A. Systematic review on the prevalence of low back pain in Nigeria. Middle East J Rehabil Health Stud. (2017) 4:e45262. 10.5812/mejrh.45262

[9] Traeger AC Henschke N Hübscher M Williams CM Kamper SJ Maher CG. Estimating the risk of chronic pain: development and validation of a prognostic model (PICKUP) for patients with acute low back pain. PLoS Med. (2016) 13:e1002019. 10.1371/journal.pmed.1002019 27187782 PMC4871494

[10] Andersson GB Pope MH Frymoyer JW Chaffin DB. Occupational low back pain. Assessment treatment and prevention. Mosby Year Book. (1977) 2:132–50.

[11] From I Wilde-Larsson B Nordström G Johansson I. Formal caregivers' perceptions of quality of care for older people: associating factors. BMC Res Notes. (2015) 8:623. 10.1186/s13104-015-1597-7 26517989 PMC4627617

[12] Galiatsatos P Nelson K Hale WD. Caring for the caregiver: Identifying the needs of those called to care through partnerships with congregations. J Religion Health. (2017) 56:946–50. 10.1007/s10943-017-0367-3 28188462

[13] Peters DH Garg A Bloom G Walker DG Brieger WR Rahman MH. Poverty and access to health care in developing countries. Ann NY Acad Sci. (2008) 1136:161–171. 10.1196/annals.1425.011 17954679

[14] Hesamzadeh A Dalvandi A Maddah BS Khoshknab F Ahmadi F. Family caregivers' experience of activities of daily living handling in older adult with stroke: a qualitative research in the Iranian context. Scandin J Car Sci. (2017) (3):515–26. 10.1111/scs.12365 27530936

[15] Abba MA Ahmad UA Maje AU Haruna AZ Ibrahim AA. Musculoskeletal pain and associated factors among informal caregivers of stroke survivors in Northwestern Nigeria. Mod Care J. (2022) 19:e123216. 10.5812/modernc-123216

[16] Yalcinkaya EY Ones K Ayna AB Turkyilmaz AK Erden N. Low back pain prevalence and characteristics in caregivers of stroke patients: a pilot study. Top Stroke Rehabilit J. (2010) 17:389–93. 10.1310/tsr1705-389 21131264

[17] Salma HU,. Prevalence of low back pain among caregivers of stroke survivors its impact on their activities of daily living: A dissertation submitted in partial fulfillment of the requirements for the degree of Bachelor of Science in Occupational Therapy. (2015). Available online at: http://library.crp-bangladesh.org (accessed September 20, 2022).

[18] Nargis N Thompson ME Fong GT Driezen P Hussain AK Ruthbah UH. Prevalence and patterns of tobacco use in Bangladesh from 2009 to 2012: Evidence from International Tobacco Control (ITC) study. PLoS ONE. (2015) 10:e0141135. 10.1371/journal.pone.0141135 26559051 PMC4641679

[19] Heuch I Hagen K Zwart JA. Association between body height and chronic low back pain: a follow-up in the Nord-Trøndelag Health Study. BMJ Open. (2015) 5:e006983. 10.1136/bmjopen-2014-006983 26078308 PMC4480023

[20] Cox MJ. Low Back Pain Mechanism Diagnosis and Treatment (6^*th*^ *ed.)*. Williams & Wilkins (1999) p. 23–25.

[21] Zhang T-T Liu Z Liu Y-Li Zhao J-J Liu D-W Tian Q-B. Obesity as a risk factor for low back pain: a meta-analysis. Clin Spine Surg. (2018) 31:22–7. 10.1097/BSD.0000000000000468 27875413

[22] Sherlock LP. Stroke in developing countries: epidemiology, impact and policy implications. Develop Policy Rev. (2010) 28:693–709. 10.1111/j.1467-7679.2010.00505.x26555122

[23] Hoy D Bain C Williams G March L Brooks P Blyth F. Systematic review of the global prevalence of low back pain. Arthr Rheumatol. (2012) 6:2028–37. 10.1002/art.34347 22231424

[24] Calmels P Béthoux F Condemine A Minon F. Low back pain disability assessment tools. Ann Readapt Med Physiol J. (2005) 48:288–97. 10.1016/j.annrmp.2005.04.008 15932777

[25] Leclaire R Blier F Fortin L Proulx R. A cross-sectional study comparing the Oswestry and Roland-Morris Functional Disability scales in two populations of patients with low back pain of different levels of severity. Spine. (1997) 22:68–71. 10.1097/00007632-199701010-00011 9122784

[26] Macedo LG Maher CG Latimer J. Responsiveness of the 24-, 18- and 11-item versions of the Roland Morris Disability Questionnaire. Eur Spine J. (2011) 20:458–63. 10.1007/s00586-010-1608-2 21069545 PMC3048224

[27] Leboeuf-Yde C LauritsennJ Lauritzen T. Why has the search for causes of low back pain largely been nonconclusive? Spine. (1997) 22:877–81. 10.1097/00007632-199704150-00010 9127921

[28] Hayes J Chapman P Young LJ Rittman M. The prevalence of injury for stroke caregivers and associated risk factors. Top Stroke Rehabilit J. (2009) 16:300–8. 10.1310/tsr1604-300 19740734

[29] Vincent-Onabajo GO Daniel H Lawan A Ali MU Masta MA Modu A. Musculoskeletal symptoms among family caregivers of community-dwelling stroke survivors in Nigeria. J Car Sci. (2018) 7:59–66. 10.15171/jcs.2018.010 29977875 PMC6029650

[30] Tong HC Haig AJ Nelson VS Yamakawa KS Kandala G Shin KY. Low back pain in adult female caregivers of children with physical disabilities. Arch Pediatr Adolesc Med. (2003) 157:1128–33. 10.1001/archpedi.157.11.1128 14609905

[31] Okuda M Umemura M Yamami N Ogihara R Mano Y Hosaka T. Study on fatigue and health disturbance in caregivers of the elderly at home. Japanese J Primary Care. (2004) 27:9–17. 10.3861/jshhe.65.282

[32] Hori Y Hoshino J Suzuki K. Physical and psychological health problems among Japanese family caregivers. Nagoya J Med Sci. (2011) 73:107–15. 21928692 PMC4831219

[33] Akosile CO Okoye EC Nwankwo MJ Akosile CO Mbada CE. Quality of life and its correlates in caregivers of stroke survivors from a Nigerian population. Qual Life Res. (2011) 20:1379–84. 10.1007/s11136-011-9876-9 21380764

[34] Vincent-Onabajo G Ali A Hamzat T. Quality of life of Nigerian informal caregivers of community-dwelling stroke survivors. Scand J Caring Sci. (2013) 27:977–82. 10.1111/scs.12017 23240860

[35] Han B Haley WE. Family caregiving for patients with stroke. Rev Analysis Stroke. (1999) 30:1478–85. 10.1161/01.STR.30.7.1478 10390326

[36] Haley WE Allen JY Grant JS Clay OJ Perkins M Roth DL. Problems and benefits reported by stroke family caregivers: results from a prospective epidemiological study. Stroke. (2009) 40:2129–33. 10.1161/STROKEAHA.108.545269 19407230 PMC2707750

[37] King RB Ainsworth CR Ronen M Hartke RJ. Stroke caregivers: pressing problems reported during the first months of caregiving. J Neurosci Nurs. (2010) 42:302–11. 10.1097/JNN.0b013e3181f8a575 21207768 PMC3064495

[38] Gbiri CA Olawale OA Isaac SO. Stroke management: Informal caregivers' burdens and strians of caring for stroke survivors. Ann Phys Rehabil Med. (2015) 58:98–103. 10.1016/j.rehab.2014.09.017 25752228

[39] Schneider S Lipinski S Schiltenwolf M. Occupations associated with a high risk of self-reported back pain: representative outcomes of a back pain prevalence study in the Federal Republic of Germany. Eur Spine J. (2006) 15:821–33. 10.1007/s00586-005-1015-2 16432750 PMC3489435

[40] Eyichukwu O Ogugua PC. Epidemiology of LBP in Enugu, Nigeria. Nigerian J Orthopaed Trauma. (2012) 11:28–37.

[41] Omokhodion FO. Low back pain in an urban population in Southwest Nigeria. Trop Doct. (2004) 34:17–20. 10.1177/004947550403400107 14959964

[42] Marras W Davis KG Ferguson SA Lucas BR Gupta P. Spine loading characteristics of patients with low back pain compared with asymptomatic individuals. Spine. (2001) 26:2566–74. 10.1097/00007632-200112010-00009 11725237

[43] Yiengprugsawan V Harley D Seubsman S Sleigh AC. Physical and mental health among caregivers: findings from a cross-sectional study of Open University students in Thailand. BMC Public Health. (2012) 12:1111. 10.1186/1471-2458-12-1111 23267664 PMC3543164

[44] Suzuki K Tamakoshi K Sakakibara H. Caregiving activities closely associated with the development of ow-back pain among female family caregivers. J Clin Nurs. (2016) 25:2156–67. 10.1111/jocn.13167 27105394

[45] Hinojosa MS Rittman M. Association between health education needs and stroke caregiver injury. J Aging Health. (2009) 21:1040–58. 10.1177/0898264309344321 19773599

[46] Hafsteinsdottir TB Vergunst M Lindeman E Schuurmans M. Educational needs of patients with a stroke and their caregivers: A systematic review of the literature. Patient Educ Couns. (2011) 85:14–25. 10.1016/j.pec.2010.07.046 20869189

[47] Oupra R Griffits R Pryor J Mott S. Effectiveness of Supportive Educative Learning programme on the level of strain experienced by caregivers of stroke patients in Thailand. Health Soc Care Commun. (2010) 18:10–20. 10.1111/j.1365-2524.2009.00865.x 19519873

[48] Faber J Fonseca LM. How sample size influences research outcomes. Dental Press J Orthod. (2014) 19:27–9. 10.1590/2176-9451.19.4.027-029.ebo 25279518 PMC4296634

[49] Rosenman R Tennekoon V Hill LG. Measuring bias in self-reported data. Int J Behav Healthc Res. (2011) 2:320–32. 10.1504/IJBHR.2011.043414 25383095 PMC4224297

